# A Patient-Centred Medical Home Care Model for Community-Dwelling Older Adults in Singapore: A Mixed-Method Study on Patient’s Care Experience

**DOI:** 10.3390/ijerph19084778

**Published:** 2022-04-14

**Authors:** Mimaika Luluina Ginting, Chek Hooi Wong, Zoe Zon Be Lim, Robin Wai Munn Choo, Sheena Camilla Hirose Carlsen, Grace Sum, Hubertus Johannes Maria Vrijhoef

**Affiliations:** 1 Geriatric Education and Research Institute, Singapore 768024, Singapore; wongchekhooi@tsaofoundation.org (C.H.W.); zoe.lim@geri.com.sg (Z.Z.B.L.); choo.robin.wm@geri.com.sg (R.W.M.C.); sheenacarlsen13@gmail.com (S.C.H.C.); sum.grace.ce@geri.com.sg (G.S.); 2 Tsao Foundation, Singapore 168730, Singapore; 3 Health Services & Systems Research, Duke-NUS, Singapore 169857, Singapore; 4 Panaxea, B.V., 1098 XH Amsterdam, The Netherlands; bert.vrijhoef@panaxea.eu; 5 Department of Patient & Care, Maastricht University Medical Center+, 6229 HX Maastricht, The Netherlands

**Keywords:** patient-centred care, patient-centred medical home, integrated care, patient experience, mixed-method, care quality

## Abstract

Patient-Centred Medical Home (PCMH) is a strategy to enhance patient-centredness to improve care experience. We aimed to understand patient experience of an integrated PCMH model for complex community-dwelling older adults in Singapore. We used a mixed-method design with a prospective single-group pre-post quantitative component and a concurrent qualitative component. Participants were administered the validated Consumer Assessment of Health Providers and Systems Clinician & Group Survey (CG-CAHPS) at baseline (N = 184) and 6-month (N = 166) post-enrolment. We conducted focus group discussions (FGDs) on a purposive sample of 24 participants. Both methods suggest better care experience in PCMH relative to usual care. There were improvements in the CG-CAHPS measures on patient–provider communication, care coordination, office staff interactions, support for patients in caring for their own health, and provider rating in PCMH relative to usual care. In the FGDs, participants reported benefits of consolidated appointments and positive experience in sustained patient–provider relationship, shared-decision making, and family/caregiver engagement in PCMH. Participants may not fully comprehend the concept of integrated care, hindering both the effective communication of the intended care model and perceived benefits such as the provision of multidisciplinary team-based care.

## 1. Introduction

Population ageing has a significant impact on healthcare systems globally with increasing prevalence of multiple chronic diseases and complex medical and psychosocial care needs. Similarly, in Singapore, there is an increasing trend of ageing population in the last decade with higher demand for managing complex care patients [1]. This necessitates health systems, including primary care, to increase coordinated strategies to provide more integrated health and social care services [2]. The lack of integration can result in poorer care experience and adverse outcomes from suboptimal follow-up and continuity of care [3,4,5,6].

The concept of patient-centredness has been identified as one of the approaches to address patients with complex needs [7]. In the literature, published definitions of patient-centredness with variations in terminologies, conceptual meaning and dimensions, stem from different theories and disciplines [8,9,10,11,12,13]. Despite the different origins, all share a fundamental approach to care that emphasises on individuals’ values and preferences in guiding various aspects of their health care, to support their health and life goals [3,11]. Recent study proposed essential dimensions of an integrated model of patient-centredness in older adults with identified priorities on seeing the patient as a unique person, patient–provider communication, patient involvement in care, physical, cognitive and emotional support, and involvement of family and friends [14].

Patient-centredness is also identified as an important domain of healthcare quality and in policy and practice developments, promoting patient-centred care across health care systems globally [8,9,15]. It aims to improve care quality with a shift from the traditional biomedical model to one that emphasises patient preferences, needs, and values [8]. This shift is accompanied by the increasing emphasis given to the importance of patient experience in healthcare assessment, as it provides insights into patients’ perception and experience of patient-centredness and reflects subjective reports of healthcare quality from patients’ perspective as patients assess different aspects of their care over time [6,8,16,17,18,19,20]. Patient experience data were also reported as actionable in making improvements in patient-centredness by both practice leaders and organisations [21,22,23,24,25]. Patient experience measures could help quality improvement planning by giving insights at which organisational processes practices must modify or adopt to achieve patient-centredness based on patients’ perspective [26].

Patient-Centred Medical Home (PCMH), a re-engineered primary care practice with a team-based accessible care model that is both proactive and coordinated, has been advocated as an important strategy to enhance patient-centredness with its goal to improve care experiences and outcomes while reducing costs [27,28,29]. Primary care was also viewed as important as this was where the majority of patients were closely connected to the health system [30]. 

In 2012, the Regional Health Systems (RHSs) were introduced by Singapore Ministry of Health with focus on integrated population health management within a defined geographical region [31,32]. Currently, care for older adults with complex needs in Singapore is provided mainly in large acute hospitals and Specialist Outpatient Clinics (SOCs), producing a significant strain on health resources [33,34]. The PCMH model is currently tested as a community-based primary care model for complex older adults, as Singapore’s RHSs are also looking at addressing their needs longitudinally in the community. Box 1 summarises the terminology used in this study.

Studies on PCMH reported positive evidences on enhanced biomedical outcomes and health-related quality of life, reduction in hospital admission, improved education and self-management support, and cost-effectiveness of PCMH as compared to standard primary care [35,36]. However, findings on patient experience outcomes were mixed [18,35,37,38,39]. Studies found variable effects on patient experience despite the evidence for the positive relationship between patient experience and key domains of the PCMH model, such as accessibility, comprehensiveness and continuity, care coordination and management of services [40,41,42,43]. This might be due to variations in PCMH practices characteristics, target population, care setting, and level of PCMH adoption [44]. The large majority of the PCMHs is a hybrid model with a combination of having an additional function of a care management (CM), while a smaller number of PCMH extends the model to service integration with social services within the practice, as well as integration with the large community and medical network where the practice resides [45]. The “integrated type” appears to have more comprehensive impacts in decreasing costs and utilisation, improving access, clinical quality, preventive services, and patient satisfaction [45]. While most studies on PCMH concentrate on improving care for chronic medical conditions, functional impairment, and mental health, only a few focus on addressing social care needs [10,35,46,47,48].

Box 1 **Terminology Used in This Study**

Regional Health Systems (RHSs): These are integrated clusters of public healthcare systems. They operate and build partnership among a range of healthcare providers within their respective region, including acute and community hospitals, primary care providers, nursing homes, and other long term care providers. Currently, there are 3 RHSs in Singapore (Central, Eastern, and Western regions). The PCMH was implemented in partnership with the Central RHS.

Specialist Outpatient Clinics (SOCs): These are clinics that provide ambulatory care and outpatient consultation for a more specific expertise or specialty. In Singapore, public-sector SOCs are located within and operationalised as part of an acute hospital, and offer services at a subsidised cost.

Bio-Psycho-Social (BPS) Risk Screener: A tool that aims to capture the biopsychosocial health risk and managing status in the context of aging. It contains 19 questions on biological health (seeing/hearing/communication, looking after yourself, getting around), 12 questions on psychological health (feeling safe, emotional well-being), and 6 questions on social health (relationships, social independence). Each of the domains contributes a score of 0, 1 or 2 (higher score reflects higher risk of poor heatlh within the domain). The total score ranges from 0 to 6, whereby scores 0 to 1 are categorised as “Managing well”, scores 2 to 3 are categorised as “Some problems”, scores 4 to 5 are categorised as “Many problems” and score 6 is categorised as “Overwhelming problems”. Patients with high bio-psycho-social health risk were those in the latter two categories.

Both integrated and patient-centred care are often identified as ideal ways to manage older adults because the nature of their diverse and dynamic needs that will benefit from these more responsive and coordinated care models [10,49,50]. There is an increasing awareness on the importance of having patient-centredness in integrated care but only few studies explore what patient-centredness means for patients in the integrated care context and how integrated care promotes patient-centred care from patients’ experience and perspective [51,52]. Moreover, studies exploring patient-centredness in integrated care are limited to experiences from countries outside Asia [15,52,53]. It is important to address this literature gap to inform further implementation and evaluation of the care model.

In this study, we evaluate patient’s experience in an integrated PCMH model in Singapore and the extent that the experiences met the target goals of PCMH care domains of comprehensiveness, patient-centredness, accessibility, and coordinated care, with a mixed-method approach. This is the first study to our knowledge that describes patient experience of a PCMH model from the patient’s perspective in Asia. Findings from this study would have important implications in addressing the knowledge gap in understanding how complex patients experience patient-centred care in integrated PCMH model in an Asian context. This study is part of a larger evaluation of the PCMH model that aims to assess its effectiveness on health services utilisation and cost, patient activation, and quality of life [36,54].

## 2. Materials and Methods

### 2.1. Programme Description

The PCMH model is a programme that provides integrated health and social care in primary care for adults with complex needs while adopting PCMH care domains (i.e., patient-centredness, comprehensive care, accessibility, coordinated care, quality, and safety) defined by the Agency for Healthcare Research and Quality (AHRQ) in its implementation. It is part of a larger community-based service delivery system that provides services to promote active ageing. Implementation of this new care model was done in close partnership between a community-based social service agency (SSA), a public hospital-based healthcare institution and a public primary care clinic in Singapore’s central region. PCMH was financed by a government subvention and funding from Tsao Foundation, which is the family foundation that funds the SSA. It was an innovative programme that used existing primary care financing as part of Singapore’s National chronic disease management programme [55], with access to financing schemes and government subsidies, such as: (i) Community Health Assist Scheme, a subsidy by the government for outpatient medical care and selected chronic conditions [56]; and (ii) Medisave, a mandatory national saving scheme, to offset patients’ out-of-pocket payment for management of selected chronic conditions [57]. In addition, patients’ out-of-pocket payment could be reduced with: (i) RHS-based medication subsidies; and (ii) the SSA’s own subsidy for patients in need who cannot afford the care despite using government subsidies and Medisave. Details on the programme description are described elsewhere [36,54].

The PCMH targeted community-dwelling, ambulant adults aged 40 and above with complex needs, residing within the geographically-defined service area. The cut-off age of 40 was chosen to reflect the life course approach, whereby PCMH services were offered to patients with complex needs from their fourth decade of life, before reaching old age, for early intervention to prevent further adverse health outcomes [58]. Complex needs were defined as having high biopsychosocial care needs based on a community risk screening tool, clinical judgement, and/or pre-existing risk stratification criteria by the partner healthcare institutions [36,54]. The screening tool used was the Bio-Psycho-Social (BPS) Risk Screener—[59]. This tool was validated in a nationally representable sample of older Singaporeans aged 60 and over.

Patients enter into PCMH programme through 3 pathways: (i) walk-ins, (ii) internal referrals from the larger community-based service delivery system implemented by the SSA (e.g., dementia care services, day centre); and (iii) external referrals from the public healthcare institution partners (acute hospital and primary care clinic) and other community-based providers. All patients were screened and triaged for complex biopsychosocial care needs using the BPS Risk Screener and clinical judgement, except for those referred by the public healthcare institutions partners that used criteria of higher risk patients as defined by their respective pre-existing programme categorisation of complex patients. For primary care clinic, complex patients are deemed as those having multimorbidity with complications, patients with dementia, or patients with psychosocial issues. Referrals from acute hospitals include patients from the geriatric medicine and internal medicine specialist clinic who were triaged based on additional assessment in psychosocial needs. Eligible patients will undergo comprehensive needs assessment within the first month of enrolment to PCMH.

**Figure 1:**
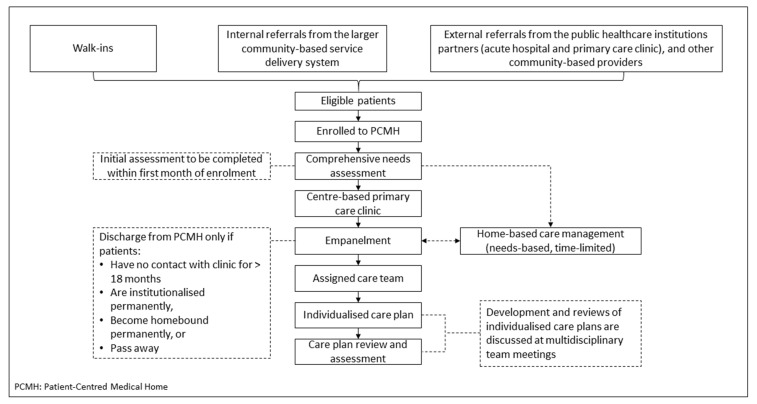
Patient Flow Diagram.

Patient care was delivered by a multi-disciplinary care-team: (i) a centre-based primary care clinic, led by family physicians trained in care for older adults and simple care coordination, and (ii) home-based time-limited care management (CM) service, led by a medical social worker or nurse care manager, given for those needing more complex psychosocial care. Figure 1 shows the PCMH patient flow diagram.

### 2.2. Study Design

This is a mixed-method study with a prospective single-group pre-post quantitative component and a concurrent qualitative component with a mixed deductive-inductive framework approach. The mixed-method design aims for triangulation (i.e., seeking convergence and corroboration of results from both methods) and complementarity (i.e., seeking elaboration, enhancement, illustration, and clarification of results from one method with results from the other method) of quantitative and qualitative data in understanding patient experience in the PCMH. The quantitative and qualitative data were collected concurrently, given equal weightage, analysed separately and integrated *after* analysis of each was completed, specifically during interpretation of the findings. The reporting of the qualitative component is guided by the Consolidated criteria for Reporting Qualitative research (COREQ) guidelines (Supplementary File S1: COREQ Checklist) [60].

This study was retrospectively registered with ClinicalTrials.gov (Protocol ID: NCT04594967), and all methods were performed in accordance with the relevant guidelines and regulations of the study registration submission. 

### 2.3. Sampling and Recruitment

**Figure 2:**
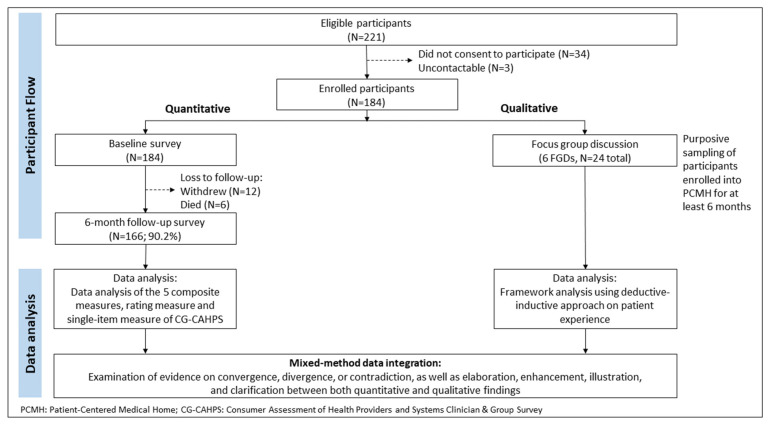
Participant Flow and Data Analysis.

Participants were recruited from November 2017 to April 2019. Eligibility criteria for recruitment were: (i) aged 40 years and above; (ii) had a high biopsychosocial health risk as defined by a 37-item Bio-Psycho-Social (BPS) Risk Screener validated in the local setting [59], or pre-existing risk stratification criteria used by referring healthcare institutes and/or clinical judgement; and (iii) resided in Whampoa, a geographically defined region in Singapore (total population of 41,000) where the PCMH was located. Figure 2 displays the participant flow.

For the quantitative component, we recruited 184 eligible participants at baseline, who completed the baseline survey. Eighteen of 184 participants were lost to follow-up, resulting in 166 participants who completed the survey at 6-month post-enrolment.

For the qualitative component, we selected a purposive sample of participants who received care from the PCMH for at least 6 months and agreed to participate in the FGD. Participants for FGDs were identified among participants who did the quantitative survey and were contacted by telephone at 6-month post-enrolment to obtain their interest in participating in the FGDs. Out of 96 eligible potential participants approached, 72 were not interested to participate in the FGDs, resulting in 24 participants recruited for 6 FGDs. The sample size was guided by the threshold of saturation, when there were no new codes, themes, or patterns emerging from the data. The first session was a pilot FGD with 2 participants. No substantial modification was done to the topic guide following the pilot FGD and we included the pilot FGD data in the analysis. The subsequent FGDs had 4 to 5 participants in each. Three FGDs had 4 participants and 2 FGDs had 5 participants.

Primary caregivers were recruited under both the quantitative and qualitative study components. They acted as proxies to represent patients who could not take part in the study due to cognitive impairment as clinically diagnosed by their attending physician. Informed consent was taken from all participants. This study was approved by the National Healthcare Group Domain Specific Review Board (NHG-DSRB) Singapore (Reference: DSRB 2017/00352).

### 2.4. Quantitative Component

#### 2.4.1. Data Collection

We administered the Consumer Assessment of Health Providers and Systems Clinician & Group Survey (CG-CAHPS) at baseline and 6-month post-enrolment. The survey collected quantitative data on patient experience on care. This instrument has been widely used to measure patient-reported care experience in the context of primary care and also validated among Asian populations in the US [56,57]. It measures patient care experience with healthcare providers and staff of the assessed practice in the 6-month prior to the survey. The instrument generates three types of measures, i.e., rating measures, composite measures, and single-item measures [58].

Rating measures are collected from items that use a scale of 0 to 10 to measure how patients assess their providers. Composite measures are derived from combining results from items that measure the same dimensions of patient experience. Single-item measures are individual questions that did not fit into composite measures. While composite and rating measures are recommended for public reporting and consumers, the single-item measures are useful to healthcare providers to identify areas for improvement [59]. We used the CG-CAHPS survey supplemented with PCMH and Access items and adapted the socio-demographic questions according to local context.

The CG-CAHPS survey was conducted face-to-face by trained surveyors at baseline, defined as within 60 days of enrolment. This assesses their care experience with their usual primary healthcare provider in the 6 months prior to enrolling into PCMH. The primary healthcare provider was defined as the main provider for the participant’s chronic care needs. The second survey was conducted at 6-month post-enrolment. This assesses patient experience with PCMH. Surveys were translated to English, Chinese, and Malay by professional translators, and conducted in the preferred language of the participant.

We used additional data sources to obtain diagnosed and self-reported physical and mental health conditions (based on International Classification 9 and 10 codes). The data sources included the PCMH and Regional Health System (RHS) administrative database and the 5-level EuroQol 5-dimension (EQ-5D-5L) survey [60].

#### 2.4.2. Data Analysis

We reported 5 composite measures (timely care access; patient–provider communication; care coordination; helpful, courteous and respectful office staff; support for patients in caring for their own health), 1 rating measure (patient’s overall rating of the provider), and 1 single-item measure (provider talking with patient about things that worried him/her, or caused him/her stress) from the CG-CAHPS. Composite measures were computed by calculating the mean score across its respective item groups [61,62]. Timely care access, patient–provider communication, care coordination, and helpful, courteous staff had response categories in a 4-point scale (“never”, “sometimes”, “usually”, “always” were scored as 1, 2, 3, and 4, respectively) [61]. Support for patients in caring for their own health had response category in a 2-point binary scale (“no” and “yes” were scored as 0 and 1, respectively) [61]. Rating measure was based on responses to a question on provider rating, with 11-point scale (0 being the worst and 10 being the best) [61]. Single-item measure was reported by calculating the percentage of participants responding “yes” to the question asking whether someone from the provider’s office talk about things in life that worried them, or caused them stress. Each item responses were adjusted with participant’s age and general health rating before its score was calculated, to account for case mix differences.

The CG-CAHPS items were only applicable to participants having the relevant care experience. For example, an item that asks about the frequency of timely appointment for urgent care was only applicable to participants who had contacted the provider for an urgent care appointment. It was a skipped question for those who did not require urgent care. Different item weights were assigned based on different number of responses for the items [61,62]. After complete data collection, one participant was found to have the baseline survey completed more than 60 days from PCMH enrolment date. We did a sensitivity analysis to exclude this participant and compare the results with the completed cohort.

Data analysis was conducted in SAS version 9.3 (SAS Institute Inc., Cary, NC, USA), using the CAHPS Analysis Program Version 4.1, which was designed to assist users in calculating the scores for all CAHPS survey measures and adjusting for case mix [61].

### 2.5. Qualitative Component

#### 2.5.1. Data Collection

A semi-structured topic guide was developed using PCMH care domains as the conceptual framework, with additional domains on perceived benefits and impacts (Supplementary File S2: Focus Group Discussion Topic Guide) [63]. FGDs were audiotaped and conducted in two languages (English and Chinese) by an independent professional facilitator at a quiet, enclosed space within the PCMH programme’s venue. The facilitator and the study team did not know the participants prior to the FGD.

FGDs were between 60 to 90 min, audio-recorded, and transcribed verbatim. Field notes were taken during the FGDs to document observations of the FGDs, including non-verbal language and interactions between participants. For FGDs conducted in Chinese, the transcripts were forward-translated to English and backward-translated to Chinese. All translated transcripts were vetted by the study team to ensure accuracy of translation.

#### 2.5.2. Data Analysis

We used the Framework method to analyse qualitative data with a mixed deductive-inductive approach [64,65,66]. First, two researchers (M.L.G., S.C.H.C.) read and familiarised themselves with the data while having the prior conceptual framework and the research objective in mind. Initial topics within the data were identified deductively according to the conceptual framework and the topic guide questions. Emerging themes were noted during this stage. Additional topics not in the original framework were added after familiarisation and constant re-visiting of the data. The final topics were then indexed or labelled in the transcript to organise the data.

Next, the indexed data were organised into thematic charts with topics arranged as column headers and the FGD groups and participants as row headers, so that individuals or each FGDs can be compared. Within each topic, we applied both structural coding and content coding. Structural coding was done by segmenting the texts based on the question and prompt asked. Content coding was done by segmenting the texts based on the meaning they exemplified [67].

Finally, theme synthesis and interpretation were done by mapping the range and nature of phenomena, finding association and explanation within and between the themes. Findings were presented to other study members and advisors for further refinement and agreement in theme interpretation. Microsoft excel and NVivo 12 (QSR International, Doncaster, Australia) were used for data management.

### 2.6. Data Integration

Figure 2 summarises the mixed-method data analysis. Findings from both parts were contrasted and compared using PCMH care domains as the conceptual framework—comprehensive care, patient-centred, accessible service, and care coordination [63]. We looked for evidence of convergence, divergence, or contradiction, as well as elaboration, enhancement, illustration, and clarification between the two components [68]. Results from qualitative FGDs were compared with quantitative survey results to validate (convergence), broaden explanatory model (divergence, contradiction), or explain (explanatory) [69]. Qualitative themes were utilised to provide deeper insights to the quantitative results. Quantitative data on participants’ profile such as disease complexity and self-reported physical and mental health provided additional context to the qualitative findings.

## 3. Results

### 3.1. Baseline Sociodemographic, Medical Conditions and Healthcare Utilisation

Table 1 presents the baseline characteristics of all the study participants (N = 184) and the participants who did the FGDs (N = 24). Median age of the participants was 79 years (IQR: 13). Majority were female (56.0%), Chinese (93.5%), married (50.0%), had no formal education (51.6%), and lived in 3-room public housing apartment (48.4%). Participants had high disease complexity as reflected by the median age-adjusted weighted Charlson Comorbidity Index (CCI) (IQR) of 4 (3) and reported a median of five chronic diseases per person with at least one of three geriatric syndromes, namely incontinence (28.8%), frequent falls (25%), and dementia (24.5%). In addition, 44.6% reported difficulty with mobility, 36.4% with usual activities, 21.2% with self-care, and 22.5% with anxiety or depression at baseline. The public primary care clinic, private primary care clinic, and hospital were reported as the primary healthcare provider prior to PCMH in 45.1%, 28.8%, and 26.1% of the participants.

Of the 24 FGD participants, 20 were patients of the PCMH and 4 were primary caregivers to patients of the PCMH. Majority (41.7%) attended the public primary care clinic as their primary healthcare provider prior to PCMH. They received care from the PCMH between 6 months to 1 year and 7 months at the time of the FGD (Table 2).

### 3.2. Quantitative Results

Participants reported better care experience in PCMH relative to their usual care (Table 3). Compared to baseline, the composite scores for patient–provider communication, care coordination, and office staff interactions improved by 8.9%, 5.3% and 9.5%, respectively. Compared to baseline, the score on the measure of support for patients in caring for their own health in PCMH versus usual care doubled from 15% to 32%. A higher proportion of participants stated that the provider talked about patient’s worries and stress in PCMH (9.04%), compared to the proportion at baseline (6.98%). We found an improvement of 15.6% on provider rating by patients under the PCMH, compared to usual care at baseline. Timely care access was not calculated due to insufficient responses for a representative composite score (responses were fewer than 100) [62]. Sensitivity analysis shows no difference when excluding the participant (n = 1) with the baseline survey done more than 60 days from PCMH enrolment date (Supplementary File S3).

### 3.3. Qualitative Results

We identified seven themes based on participants’ description of their care experience. The seven themes were (i) PCMH as a “specialised” primary care model, (ii) consolidated appointments within PCMH, (iii) attention to psychosocial care needs, (iv) relationship-based care and shared-decision making, (v) engaging family and caregivers as partners in patient care, (vi) enhanced access for better continuity of care, and (vii) inherent integration between PCMH and different healthcare providers through linked medical records. We mapped the seven themes into the PCMH care domains. One theme was categorised as a context as it did not fit into the PCMH care domains.

#### 3.3.1. Context

##### PCMH as a “Specialised” Primary Care Model

There were differing understandings of the care model as participants compared the PCMH to their usual care. Comparisons were made based on the attending physician’s specialty, charges and subsidies, target patient population, and location of service. Participants recognised PCMH as most similar with existing primary care models as it was served by primary care physicians, situated in a similar setting (i.e., the community), and lacking supporting facilities usually available only in a hospital setting.

*FGD4P3: “From what I see, the doctors from [PCMH] are **not specialist**…[PCMH] feels like it’s a **general doctor**…”*

*FGD4P1: “Even if you call for that [specialist] doctor here, [PCMH] is still a normal clinic because they do not have the equipment…they will only have it at other hospitals…it is exactly the same as the polyclinic [i.e., public primary care clinic]”*

Participants’ understanding of PCMH was also influenced by the information received upon referral to PCMH. In most cases, participants were not fully informed of the programme identity, whether it had any affiliation with the referring institution, or a charity-based service run by an SSA.

*FGD1P1: “…is it [PCMH] have their own doctors or they actually get their doctors from somewhere, **the doctors are doing voluntary work**. So they come in on certain days and others they don’t come in at all. So, I really don’t know, how is the place, how uh, **how the organization is structured**…”*

*FGD1P1: “No [there was no introduction about PCMH when referred]. So they [public acute hospital] told us that **they have a similar clinic** in [name of region omitted] and uh, whether you want to have a try…”*

However, participants perceived the differentiating characteristic of PCMH as a “specialised” primary care model because it targeted mainly older adults with complex care needs, e.g., those with chronic diseases and frailer with functional disabilities, living within the geographically defined region.

*FGD4P3: “Frankly speaking, it [PCMH] is **different from the other polyclinics** [public primary care clinic]. This is **more specialised for elderly**…”*

*FGD4P3: “Yes, cannot visit…you have to exceed a certain age, like 40 years, then you can. It is different, they only attend to the elderly…it **does not work like the clinic outside**.”*

*FGD4P2: “For chronic illness. They [PCMH] **attend to chronic diseases**.”*

#### 3.3.2. PCMH Domain: Comprehensive Care

##### Consolidated Appointments within PCMH

Participants reported a perceived benefit of having consolidated appointments within PCMH for follow-up care and medication refills for their chronic conditions.

*FGD1P1: “…previously uh we go to a few medical providers. So now the primary care provider actually is [PCMH]. So because of [PCMH], I think her **appointment with other doctors have reduced**.”*

This was viewed as an additional value on convenience as compared to the multiple appointments to different healthcare providers they experienced prior to PCMH. Participants with their care transferred to PCMH perceived the same degree of care as their previous providers, as they could obtain their regular medications and similar follow-up care. The option to return to their specialists, should their conditions deteriorate, also contributed to participants’ confidence in care transfer to PCMH.

*FGD2P1: “My mother comes here [PCMH] because she is seeking medical advice for the same sickness like she did in [name of public primary care clinic omitted]…If there is no emergency, then **it is the same** [care for chronic disease], even the report is the same. The medicines are also the same. Then **unless if there is anything, like my mother has water in her lungs**, I tell him, then, he gives me medication. **If she doesn’t get well after taking the medication,** I will ask him to refer me back to [name of public acute hospital omitted].”*

##### Attention to Psychosocial Care Needs

Participants appreciated the attention on social health given by PCMH, who encouraged them to socialise in the PCMH’s shared community space, offered companionship and home-based therapy for the less active patients, and provided caregiver support. In addition, they also appreciated the access, attention, and support given for their psycho-emotional needs by the home-based CM service.

*FGD5P4: “He [PCMH doctor] asked me to come here [PCMH shared community space], to come over whenever I am free, to not stay alone at home.”*

*FGD2P2: “Actually my mum trial here [day-care] for two weeks…day-care, but she doesn’t like it. So they’re [PCMH] very good. They assign someone [referring to care manager] to go over to her house once a week for one hour. Just to talk to her, accompany her, do some therapy.”*

However, participants were unable to identify the care managers as part of their care team, even for those receiving the service. The care managers were identified as volunteers.

*FGD3P3: “He [mentioned as social worker—referring to CM] only checks in on us [caregiver and the PCMH patient], see if we can do certain things. He asked me if I [caregiver] can accept [caregiving duty]. Can I do this properly? Like that. He is comforting me.”*

#### 3.3.3. PCMH Domain: Patient-Centred

##### Relationship-Based Care and Shared-Decision Making

Participants appreciated relationship-based care that sought to understand their needs, respect their preferences in care planning, and treat them holistically as a person, rather than as a disease. When comparing with their previous providers, most participants perceived a more sustained relationship with PCMH through having access to the same provider over time, which enabled them to have better familiarity and trust with the provider. This was also supported by the sufficient consultation time, which was perceived to be one of the key differentiating factors of the PCMH.

*FGD1P1: “...because for polyclinic [public primary care clinic] and for family doctor, the doctor always changes. Even though they have the case information inside there,,,many times I heard the doctor say ‘oh, the doctor said this’…..**normally they** [doctor from other primary care clinic] **treat the condition.** So, so, **they don’t care**, for example, I went to the family doctor, I say, wah, leg swell, furosemide. Whereas when I come to [PCMH], I say leg swell, the doctor says, eh, uh why, uh, what happens, then we will explain. One of the things about uh, uh, ageing patients, I don’t think you treat the condition, they take a lot of medicine. **They [PCMH] treat the patient**…”*

*FGD3P3: “It is just the same few people [in PCMH]. So we become familiar and hence it’s easier to talk to them…you become familiar after numerous visits mah [expression for ‘rite’]? Once you are familiar **you can tell him anything**.”*

Participants described their prior providers as lacking in patience and friendliness in their communication, probably happening when the clinic was busier with a bigger crowd. This was described as a negative experience which might affect the patient–provider relationship.

*FGD6P2: “…Very friendly here [PCMH], like friends like that…they [public primary care clinic] are really busy. It was so busy ah, they have no choice but to lose their temper sometimes.”*

*FGD6P4: “…because there are many patients there, so sometimes they are busy with this, sometimes busy with that. Really, sometimes they are overwhelmed.”*

Positive experience in communication was also related to the provider’s ability to speak in patients’ preferred language. This encouraged participants to be more open when sharing about their unique needs and to better describe their conditions.

However, different preferences for patient engagement were observed. One participant expressed dislike for being asked whether she knew about her medication and its purpose. This was perceived as inappropriate due to the perception that the provider was supposed to assume the sole role in giving orders and directions for the care.

*FGD4P4: “…he [PCMH doctor] asked if I knew what medicine I was eating. I told him **if I knew, I would have been a doctor**…**Normally when you visit the doctor**, they will not allow you to go in directly. He will take about 10–20 min to study your case, then we will ask you in. They usually do. The doctor’s [PCMH doctor] way of handling…like they are not prepared, and still asked me.”*

##### Engaging Family and Caregivers as Partners in Patient Care

Participants, especially the caregivers, valued health education, which enabled better understanding of health conditions and caregiving needs, ways for self-monitoring and self-management at home. Participants also appreciated the provision of health education to foreign domestic workers (FDW) who cared for the patients, because it improved the FDW’s knowledge in caregiving. PCMH’s involvement of caregivers and family members in care planning was perceived as a good experience, as it contributed to better patient management at home.

*FGD1P1: “…every time I come here [PCMH], it’s an education process and the discussion and information given by the doctor actually it helps you understand the condition better, so you manage the patient very much better.”*

#### 3.3.4. PCMH Domain: Accessible Services

##### Enhanced Access for Better Continuity of Care

Better access was shared in relation to the geographical proximity between the clinic and patients’ home, resulting in convenient transport and travel arrangement. In addition, the clinic waiting time was much shorter as compared to their usual care experience. This was perceived to be important especially for older adults who may not tolerate long wait times.

*FGD2P1: “Not convenient [public primary care clinic]. This place [PCMH] is different. **It is just opposite my house**…appointment at 8.00, I reached at 7.45 and it took only a while to get it done. So she can go back and have her breakfast. She doesn’t need to wait. This is good for the elderly and good for us too.”*

Mixed preferences were reported regarding access to urgent care outside of office hours and during weekends. Caregivers in particular expressed their preference on having access to medical services in times of crisis, emphasising on their frustration on the unavailability of urgent care from the same provider that resulted in unnecessary visits to emergency department and discontinuity of care. However, some other participants felt that although access to urgent care was a good option, they rarely needed it and could find alternative options when needed.

Different experiences and understandings were also shared regarding access to same-day unscheduled appointments. Most understood PCMH as either non-accessible for same-day unscheduled appointments or accessible with long waiting time.

Participants perceived the importance of access to both urgent and same-day unscheduled appointments for better continuity of care, as their expectation was to have the same provider for all their care needs, including acute problems. One caregiver shared her appreciation towards a PCMH doctor who followed-up on her after an unsuccessful attempt to see the doctor on the same day during an acute episode. The participant shared the assurance she felt by having PCMH as the main provider, who is informed of all the care received from somewhere else.

*FGD1P1: “…I was thinking that it would be good [to get urgent care access]. You know, even though another doctor from the same clinic, so at least they are from the same, same organisation they are about to keep each other informed…then you don’t lose the information of what happen with the patient, what medicine is given to the patient.”*

#### 3.3.5. PCMH Domain: Coordinated Care

##### Inherent Integration between PCMH and Different Healthcare Providers through Linked Medical Records

The aspects on care coordination across different healthcare providers and referral processes received little comments from the participants. However, participants perceived an inherent integration between PCMH and the SOCs within the public sector as evidenced from the linked medical record. This gave them the reassurance that PCMH was well informed of their healthcare needs.

*FGD2P4: “He [PCMH doctor] entered my mum’s data inside, so he says this doctor [from another institution] will be able to see. So in that way, the convenient, **the connection is already there**.”*

### 3.4. Data Integration

Table 4 shows the matrixed data integration. We found evidences of convergence, elaboration, and expansion between quantitative and qualitative data. In the comprehensive care domain, quantitative data reported a higher number of participants (9.04%) with provider that talked about patients’ worries and stress in PCMH as compared to usual care (6.98%). This finding converged with what was found in qualitative data within the “attention to psychosocial care needs” theme, where participants appreciated the attention given to and access for psychosocial care in PCMH and perceived this as a positive experience. It was elaborated in the qualitative data that psychosocial care was mainly given by the home-based care management service. As most participants (64.1%) received clinic service only, this might lead to perceived absence of this psychosocial attention for those not receiving home-based care management service, reflected by the high number of participants (91%) who stated that their provider in PCMH did not talk about patients’ worries and stress in the quantitative component. Another plausible explanation is the lack of identification of the home-based care management service as part of the care team, even for those receiving the services. This was found in qualitative data. The qualitative theme of “consolidated appointments within PCMH” expanded the understanding of patient experience on comprehensive care domain, perceived as a benefit of PCMH as compared to usual care.

Data integration on other PCMH care domains is detailed in Table 4.

## 4. Discussion

This study describes how complex community-dwelling older adults experienced care provided by an integrated PCMH model in Singapore. We used a mixed-method design with a prospective pre-post without controls quantitative component and a concurrent qualitative component. Participants were older, a mean age of 77.5 years, with complex biopsychosocial needs from multiple comorbidities, functional limitations, and mental health and social needs.

Our quantitative findings suggest a better patient experience in the PCMH as compared to usual care. We found improvement in all CG-CAHPS composite measures, with better provider ratings for PCMH. The qualitative component also found more favourable care experience in PCMH as compared to usual care. No indication for divergence or contradiction was found during the quantitative and qualitative data integration.

The mixed-method approach revealed a better experience of patient-centredness in PCMH as compared to usual care, with positive experience in patient–provider relationship, person-centred communication, care personalisation, and support in managing their own health. Similar with findings from other patient-centred care studies, the qualitative findings showed that patient-centredness was defined mainly by the perceived quality of patients’ interaction with their care team [17,53,70]. Care was perceived to be patient-centred if they were seen and treated as a whole-person by a provider who understands their needs, respects their preferences, listens to their concerns, and engages them in care planning.

There were differing levels of expectations on access to urgent care. CG-CAHPS measure on timely care access was not reported due to insufficient responses in items contributing to the score, which corroborated with the qualitative findings on perceived lack of relevance for access to urgent care. It was viewed as relevant mostly by caregivers of patients with supposedly higher need for urgent care. Meanwhile, other participants felt that it was a good option but not essential. This might be related to the lower perceived urgency and complexities of care needed when in a crisis, and also the availability of alternative options in urgent care.

Patients’ complex needs required care across multiple healthcare providers and services, including medical, rehabilitative, and psychosocial. Comprehensive care provided by PCMH allowed patients to benefit from transfer of chronic care from their previous providers and psychosocial care from CM. The perceived care consolidation reflects clinical integration of previously fragmented care delivery. Previous studies noted that clinical integration tends to be a disease-focused approach rather than a person-focused approach [71]. However, with growing numbers of complex needs patients, clinical and social service integration should be complemented by a patient-centred perspective. The multidisciplinary team-based care with integrated centre-based primary care clinic and home-based CM adopted in the PCMH was part of the integration effort intended to provide comprehensive health and social care to the patients.

Appreciation on the access for psychosocial care in PCMH was reported from the qualitative findings. Although a similar finding from CG-CAHPS suggests better experience on the attention towards psychosocial health in PCMH as compared to usual care, a high number of participants (91%) stated that they did not feel like someone from PCMH had talked to them about things related to their worries and stress. The finding that most participants (64%) were receiving only clinic service might contribute to the perceived absence of this psychosocial attention which was delivered mainly by the CM service. Another plausible explanation would be the participants who received CM service could not relate their experience of receiving this psychosocial attention from CM with their overall experience with PCMH when answering the quantitative survey, as they did not perceive care managers as part of their care team.

It was noted that the qualitative findings describing patient perception on the patient–provider relationship mostly refer to the one-on-one encounter with their family physicians. The lack of understanding of the team-based model was evident from participants’ lack of recognition of home-based CM as part of their care team. A paternalistic approach may not prioritise patients’ understanding on the roles of the care team members over the goal of addressing patients’ needs. However, as patient-centred care aims to place the patients as active care recipient and partner in care planning, an aligned understanding in the care model is essential for patients to interact with care team members and to navigate for the available resources needed in their care.

Patient-centred care supports patients to be engaged in decisions about their healthcare and empowers patients to take ownership of their own health [70]. In the more traditional paternalistic model, physicians hold the main role in care planning with low patient involvement [70], whereas in a patient-centric model, the emphasis is on a more symmetrical partnership between patient and their care team in shared care planning [63]. Studies suggest that both patient-centred and paternalistic approaches can be compatible. The relationship approach can be dependent on the subject of care, especially for older adults with declines in functional and cognitive ability that might influence their “readiness” to be involved in shared decision making [15,72]. In this study, we found mixed preferences regarding patient engagement with some expressed appreciation towards more proactive patients’ role, and others did not. While this might be related to the different complexities of care needs, it could also be due to their prior experience in a predominant traditional paternalistic model. This highlights the importance of understanding and respecting patients’ interests and preferences on the role they wish or are able to play when aiming for an effective patient engagement [73,74,75].

Patient experience is a multidimensional construct which includes any process apparent to patients, e.g., subjective experience, objective experience, and observation of provider or staff behaviour [17]. The reception of experience and patient expectation would then build patient perceptions [17]. In this study, the perceived experiences in the qualitative part were observed to be a product of interaction between their previous experience and understanding in usual care with the expectation and reception of the current service encounter. Participants compared PCMH with pre-existing services in Singapore healthcare system, i.e., public and private primary care clinic or SOCs within the public sector. While these were valid comparisons as they reflected the way patients understood the transfer of care from their prior primary healthcare provider, it also resulted in a difficulty for characterisation of the PCMH as an integrated community-based health and social care model that had not been encountered by patients before. This affects the perceived experience as some intended benefit from aspects of the care model, e.g., multidisciplinary team-based care, might not be realised by the participants due to the lack of understanding. Nonetheless, majority of participants recognised the unique feature of the PCMH as a “specialised” primary care model for older adults with complex care needs.

Studies reported challenges in the accurate measurement for patient experience due to various characterisation of the terms used [17]. Patient experience is sometimes used interchangeably with patient satisfaction and as a derivative of patient perceptions [18,21,76]. Patient experience is a complex, multidimensional concept influenced by patients’ characteristics that may not be easily measurable, such as prior expectations, preferences, attitudes, and available resources, which may have been already in place before a patient ever interacts with a provider [21].

A key strength of this study was the use of a mixed-method approach that gives a comprehensive understanding of patient experience, particularly in an intervention that operates through complex causal pathways [68,77]. Triangulation and complementarity from both methods were observed, which further reinforced the strength of the findings. The value of the mixed-method here was not merely in replicating evidences generated by each approach, but also in extending understanding and adding breadth and depth to the analysis that neither method alone can offer [65,78]. Additionally, our study contributes to the literature by investigating the impact of PCMH on patient experience using a validated instrument in an Asian context.

However, there are a few study limitations. First, CG-CAHPS was initially developed to compare performance between health plans in the US and to measure practice improvement using a pre-post methodology [38,79]. However, we applied CG-CAHPS to compare two different patient experiences, i.e., in PCMH and in usual care. Second, randomisation was not feasible, which limited our study design to a single-group prospective pre-post evaluation. While we did not have a control group, this study still contributed by revealing that a complex intervention with health and social integration in primary care could improve patient experience. Additionally, our mixed-method approach showed consistent results and convergence between quantitative and qualitative component. We hypothesise that our results are biased towards the null, whereby a randomised study would show greater patience experience from PCMH, as high-risk controls would have poorer health outcomes and experiences. Third, the baseline CG-CAHPS survey was done within 60 days after programme enrolment, whereby the benefits of PCMH on patient experience may have already taken place. Hence, our findings on patient experience may be biased towards the null. Fourth, there may be self-reporting errors in this study on patient-reported experience measures. However, the questions in our study were explicitly designed to ask care experience in usual care prior to PCMH at baseline, to minimise these biases. Fifth, we included older participants with more complex needs, which may limit the generalisability of our findings to a more diverse population. Moreover, we also had non-participation from the potential participants in the qualitative component, which may introduce selection bias, whereby those who did not participate may have different experience than those interviewed. Future research may consider including participants with different level of care needs to fully capture the differing expectations and experiences. Subsequent in-depth interviews for different patient typology could also give more understanding to the context and mechanism of care experience. Sixth, the BPS Risk Screener has only been validated locally. However, since its validity scores were found satisfactorily and the current study concerns an older population in Singapore, we are confident about its psychometric merit. Further study is needed to examine the validity and reliability of the tool in different context and population. Lastly, the short follow-up period in this study might limit understanding and perceived care experience, as the impact of the PCMH may not be felt by patients until practice is close to full implementation of the model. Studies suggest that measurable patient-level results might be limited until the PCMH is fully integrated as a system of care in the unit of practice [18]. Perception in the attributes of PCMH may change with increasing awareness and longer experience with this type of care as the programme matures.

## 5. Conclusions

This study presents findings on patient experience in an early PCMH model demonstration after 6 months to 1.5 years of PCMH services within a context of a predominantly traditional doctor–patient relationship model. Both methods suggest a positive patient experience of PCMH, with better experience reported for patient–provider relationship and communication, shared decision making and family/caregiver engagement, care coordination, access and self-management support as compared to their usual care. Integrated care as a concept might not be understood and recognised fully by the participants, hindering the effective communication of the intended care model and perceived benefit from certain aspects such as the multidisciplinary team-based care. However, perceived benefits on the consolidated appointments and continuity of care, which were the products of the integration effort, were reported. Effective patient engagement and ongoing education of the intended care model are essential, as we move towards a more person-centred perspective which puts the patients in a more proactive role in care delivery.

## Figures and Tables

**Table 1:** Baseline sociodemographic characteristics of study participants.

Variable	Overall Study—Quantitative Participants (N = 184)	Qualitative Participants (N = 24)
**Age at enrolment, years, mean (SD)**	78 (9.9)	76.6 (8.6)
**Age at enrolment, years, median (IQR)**	79 (13.0)	78 (14.0)
**Female, n (%)**	103 (56.0)	16 (66.7)
**Ethnicity, n (%)**		
Chinese	172 (93.5)	24 (100)
Malay	4 (2.2)	0 (0.0)
Indian	7 (3.8)	0 (0.0)
Others	1 (0.5)	0 (0.0)
**Marital status, n (%)**		
Single	15 (8.2)	2 (8.3)
Married	92 (50.0)	11 (45.8)
Widowed	62 (33.7)	9 (37.5)
Divorced	15 (8.2)	2 (8.3)
**Education level, n (%)**		
No formal education	95 (51.6)	8 (33.3)
Primary school	53 (28.8)	9 (37.5)
Secondary school	24 (13.0)	3 (12.5)
Post-secondary (non-tertiary) and above	12 (6.5)	4 (16.7)
**Housing type, n (%)**		
***Smaller housing type***		
1–2 room public housing apartment	21 (11.4)	1 (4.17)
3 rooms public housing apartment	89 (48.4)	11 (45.8)
***Larger housing type***		
4 rooms public housing apartment	50 (27.2)	6 (25.0)
5 rooms and above public housing apartment/private housing	23 (12.5)	6 (25.0)
**Disease complexity**		
Age-adjusted Charlson Comorbidity Index, mean (SD)	4.8 (2.2)	4.3 (2.3)
Age-adjusted Charlson Comorbidity Index, median (IQR)	4.0 (3.0)	3.5 (3.0)
Number of self-reported chronic conditions, mean (SD)	5.2 (2.3)	4.8 (2.1)
Number of self-reported chronic conditions, median (IQR)	5.0 (3.0)	4.0 (3.0)
**Self-reported physical and mental health, %**		
Had difficulty with mobility	44.6	20.8
Had difficulty with usual activities	36.4	20.8
Had difficulty with self-care	21.2	16.7
Had anxiety and depression	22.5	25
**Primary healthcare provider prior to PCMH (usual care), n (%)**		
Public primary care clinic	83 (45.1)	10 (41.7)
Private primary care clinic	53 (28.8)	8 (33.3)
Hospital	48 (26.1)	6 (25.0)
**Type of service received in PCMH**		
Clinic only	118 (64.1)	19 (79.2)
Care management only	9 (4.9)	1 (4.2)
Clinic and care management	57 (31.0)	4 (16.7)

SD: standard deviation. IQR: interquartile range. PCMH: patient-centred medical home.

**Table 2:** Qualitative participants’ profile.

Study ID	Person Interviewed	Primary Healthcare Provider Prior to PCMH	Time in Programme	Type of Service Received from PCMH	Age-Adjusted Weighted Charlson Comorbidity Index	Total Self-Reported Chronic Conditions
FGD1P1	Caregiver	Private primary care clinic	7.5 months	Clinic	6	7
FGD1P2	Patient	Hospital	1 year and 2 months	Clinic and care management	3	7
FGD2P1	Caregiver	Public primary care clinic	7.5 months	Clinic and care management	8	9
FGD2P2	Caregiver	Hospital	1 year	Clinic and care management	5	7
FGD2P3	Patient	Private primary care clinic	8 months	Clinic	3	5
FGD2P4	Caregiver	Public primary care clinic	7.6 months	Clinic	10	10
FGD3P1	Patient	Hospital	6.8 months	Clinic	5	5
FGD3P2	Patient	Hospital	8.6 months	Clinic and care management	2	8
FGD3P3	Patient	Public primary care clinic	6.8 months	Clinic	7	7
FGD3P4	Patient	Hospital	1 year and 7 months	Clinic	3	6
FGD4P1	Patient	Public primary care clinic	7.4 months	Clinic	2	7
FGD4P2	Patient	Hospital	7 months	Clinic	3	5
FGD4P3	Patient	Private primary care clinic	8 months	Clinic	4	3
FGD4P4	Patient	Public primary care clinic	6.8 months	Clinic	3	5
FGD4P5	Patient	Public primary care clinic	7.8 months	Clinic	2	10
FGD5P1	Patient	Private primary care clinic	8 months	Care management	5	3
FGD5P2	Patient	Private primary care clinic	7.2 months	Clinic	5	4
FGD5P3	Patient	Public primary care clinic	11 months	Clinic	2	4
FGD5P4	Patient	Private primary care clinic	10.8 months	Clinic	3	5
FGD6P1	Patient	Private primary care clinic	11.3 months	Clinic	2	3
FGD6P2	Patient	Public primary care clinic	10.7 months	Clinic	8	4
FGD6P3	Patient	Public primary care clinic	8.9 months	Clinic	4	4
FGD6P4	Patient	Public primary care clinic	1 year and 4 months	Clinic	6	6
FGD6P5	Patient	Private primary care clinic	11.4 months	Clinic	2	5

PCMH: patient-centred medical home.

**Table 3:** Comparison of Consumer Assessment of Health Providers and Systems Clinician & Group Survey (CG-CAHPS), Adjusted Mean Composite Scores between usual care and PCMH.

Measures	Usual Care (N = 184), Adjusted Mean Score ^1^	PCMH (N = 166), Adjusted Mean Score ^2^	Percentage Difference Relative to Usual Care ^3^
**Composite measures**			
*Timely care access ^4^*—3 questions asking about patient’s experience to get timely appointments for urgent and routine care needs, and timely answers to medical questions	-	-	-
*Patient-provider communication ^5^*—4 questions asking how often providers explained things clearly, listened carefully to, showed respect for and spent enough time with patient	3.61	3.93	8.87%
*Care coordination ^6^*—3 questions asking how often provider seemed to know patient’s medical history, followed-up to give results of blood test, and asked about all the prescription medications being taken	3.62	3.81	5.31%
*Helpful, courteous, and respectful office staff ^7^*—2 questions asking how often office staff were helpful and treated patient with courtesy and respect	3.56	3.90	9.47%
*Support for patients in caring for their own health ^8^*—2 questions asking whether someone from provider’s office discussed with patient his or her specific goals for health and whether there were things in life that make it hard for patient to take care of his or her health	0.15	0.32	106.30%
**Rating measure**			
*Patient’s overall rating of the provider ^9^*—1 question asking patient to rate his or her provider on scale of 0 (the worst provider possible) to 10 (the best provider possible)	7.26	8.40	15.64%
**Single-item measure ^10^ (%)**			
1 question asking whether someone from provider’s office talk about things in life that worried the patient, or caused him or her stress	6.98	9.04	29.51%

^1,2^ The CAHPS Analysis Program adjusted the survey data for participant’s age and general health rating before the score was calculated, to account for case-mix difference. ^3^ Relative difference = (PCMH adjusted mean score—usual care adjusted mean score)/usual care adjusted mean score. Significance testing of the relative difference was not done, and the difference between scores should be interpreted descriptively. ^4^ Fewer than 100 responses, score not analysed. ^5,6,7^ Each question has response categories in a 4-point scale—never, sometimes, usually and always, scored as 1, 2, 3 and 4, respectively. ^8^ Each question has response category in a 2-point binary scale—no and yes, scored as 0 and 1, respectively. ^9^ Question is based on response with a 11-point scale—0 to 10. ^10^ Question has response category in a 2-point binary scale—no and yes. Percentage was calculated for those answering yes to the question. PCMH: patient-centred medical home.

**Table 4:** Matrixed Data Integration.

PCMH Domains	Method 1 (QUAN), Composite, Rating and Single-Item Measures	Method 2 (QUAL), Themes	Relationship	Interpretation/Comments
Comprehensive care ^1^	Higher single-item measure on question asking whether someone from provider’s office talk about things in life that worried the patient, or caused him or her stress in PCMH as compared to usual care	Attention to psychosocial care needs	Convergence and elaboration	Convergence—both quantitative and qualitative findings reported more attention given to and access for psychosocial care in PCMH as compared to usual care. Elaboration—despite the higher single-item measure in PCMH about psychosocial attention as compared to usual care, high percentage of participants stated that their provider in PCMH did not talk about patients’ worries and stress in the quantitative component. Qualitative findings elaborate the plausible explanation on the cause of perceived absence of psychosocial care reported in quantitative findings.
	No comparable quantitative finding	Consolidated appointments within PCMH	Expansion	Expansion—qualitative theme gave fuller understanding of care experience on comprehensiveness of care, particularly in consolidation of care, perceived as a benefit of PCMH as compared to usual care.
Patient-centred ^2^	Higher composite measure on patient–provider communication, office staff interaction, and support for patients in caring for their own health in PCMH as compared to usual care	Relationship-based care and shared-decision making	Convergence and elaboration	Convergence—both quantitative and qualitative findings suggest a better experience of patient-centeredness in PCMH as compared to usual care. Elaboration—qualitative component reported that whole-person orientation, sustained patient–provider relationship, provider’s attitude, communication, longer contact time during consultation, and perceived familiarity contributed to the experience on patient-centeredness in PCMH.
	No comparable quantitative finding	Engaging family and caregivers as partners in patient care	Expansion	Expansion—qualitative theme gave fuller understanding of care experience on patient-centeredness, particularly in family and caregiver education and involvement in care planning, perceived as a benefit of PCMH as compared to usual care.
Accessible services ^3^	Fewer than 100 participants responded that the questions were applicable to them—no composite measure	Enhanced access for better continuity of care	Elaboration	Elaboration—few responses in quantitative findings on the timely care access measure might be due to the experience being not relevant or applicable to them, as elaborated in the qualitative findings on their experience on urgent care and same-day appointment. Better access in terms of shorter waiting time and geographical proximity was reported in qualitative findings.
Coordinated care ^4^	Higher composite mean score of provider’s use of information to coordinate patient care in PCMH as compared to usual care	Inherent integration between PCMH and different healthcare providers through linked medical records	Convergence	Convergence—both quantitative and qualitative findings suggest a perceived better care coordination and inherent integration in PCMH.

^1^ Comprehensive care: the ability of the provider to fulfil patients’ needs (physical, mental, and social), the comprehensiveness of the range of services given within the programme, including prevention and wellness, acute care, and chronic care. ^2^ Patient-centred: describes the perceived relationship between the patient and healthcare provider, with emphasis on the whole-person orientation, respect towards patient/family preference, unique needs, culture, and values; and involves them in treatment and care. ^3^ Accessible service: provider being able to deliver accessible services with shorter waiting times for urgent needs, enhanced in-person hours, around-the-clock telephone or electronic access to a member of the PCMH care team, and alternative methods of communication such as email and telephone. ^4^ Coordinated care: experiences and perceptions on the coordination of care across different healthcare providers, including specialty care, hospital, home health care, and community services and supports, especially during transition between sites of care. PCMH: patient-centred medical home.

## Data Availability

The data presented in this study are not publicly available due to the privacy rights of the participants, in accordance with the informed consent of this study.

## Supplementary Materials

The following supporting information can be downloaded at: https://www.mdpi.com/article/10.3390/ijerph19084778/s1, Supplementary File S1: COREQ Checklist; Supplementary File S2: Focus Group Discussion Topic Guide; Supplementary File S3: Sensitivity analysis table.
