# Supplementary material for: A Patient-Centred Medical Home Care Model for Community-Dwelling Older Adults in Singapore: A Mixed-Method Study on Patient’s Care Experience
Source: Int J Environ Res Public Health. 2022 Apr 14;19(8):4778. doi: 10.3390/ijerph19084778 (PMC9030670; doi:10.3390/ijerph19084778)
Supplement: Supplementary file 1 [file ijerph-19-04778-s001.zip › Supplementary File S1.COREQ checklist.pdf]

Supplementary File S1. Consolidated criteria for reporting qualitative research (COREQ): a 32-item checklist

**No**	**Item**	**Guide questions/description**	
**Domain 1: Research team and reflexivity**			
Personal Characteristics			
**1**	Interviewer/facilitator	Which author/s conducted the interview or focus group?	Focus groups were conducted by an independent professional facilitator due to language barrier.<br><br>M.L.G. and Z.Z.B.L. vetted the forward and backward translation of the transcripts.<br><br>M.L.G. and S.C.H.C. coded and did formal analysis.
**2**	Credentials	What were the researcher's credentials? *E.g. PhD, MD*	Facilitator (B.Sci); M.L.G. (MD, MPH); Z.Z.B.L. (B.Pharm, PhD); S.C.H.C. (MSc)
**3**	Occupation	What was their occupation at the time of the study?	Facilitator (Senior Research Analyst from the consulting company); M.L.G. (Research Associate); Z.Z.B.L. (Research Fellow); S.C.H.C. (Research Officer)
**4**	Gender	Was the researcher male or female?	Facilitator (female); M.L.G. (female); Z.Z.B.L. (female); S.C.H.C. (female)
**5**	Experience and training	What experience or training did the researcher have?	Facilitator, M.L.G., and Z.Z.B.L had qualitative research training and had done previous qualitative projects; S.C.H.C. had done MSc in Global Health and Development and received qualitative training prior to conducting the analysis.
Relationship with participants			
**6**	Relationship established	Was a relationship established prior to study commencement?	No
**7**	Participant knowledge of the interviewer	What did the participants know about the researcher? e.*g. personal goals, reasons for doing the research*	Reasons for doing the research were described prior to the focus groups.
**8**	Interviewer characteristics	What characteristics were reported about the interviewer/facilitator? e.g. *Bias, assumptions, reasons and interests in the research topic*	Reasons for doing the research and interest in the research topic.

**Domain 2: study design**			
Theoretical framework			
**9**	Methodological orientation and Theory	What methodological orientation was stated to underpin the study? *e.g. grounded theory, discourse analysis, ethnography, phenomenology, content analysis*	Content analysis with framework approach.
Participant selection			
**10**	Sampling	How were participants selected? *e.g. purposive, convenience, consecutive, snowball*	Purposive
**11**	Method of approach	How were participants approached? e.*g. face-to-face, telephone, mail, email*	Telephone
**12**	Sample size	How many participants were in the study?	24
**13**	Non-participation	How many people refused to participate or dropped out? Reasons?	72 were not interested to participate in the focus groups. None dropped out.
Setting			
**14**	Setting of data collection	Where was the data collected? e.*g. home, clinic, workplace*	At a quiet, enclosed space within the PCMH program's venue.
**15**	Presence of nonparticipants	Was anyone else present besides the participants and researchers?	No
**16**	Description of sample	What are the important characteristics of the sample? *e.g. demographic data, date*	Characteristics of the sample are reported in the *Sampling and recruitment* section
Data collection			
**17**	Interview guide	Were questions, prompts, guides provided by the authors? Was it pilot tested?	Yes
**18**	Repeat interviews	Were repeat interviews carried out? If yes, how many?	No
**19**	Audio/visual recording	Did the research use audio or visual recording to collect the data?	Data was audio recorded.
**20**	Field notes	Were field notes made during and/or after the interview or focus group?	Yes

**21**	Duration	What was the duration of the interviews or focus group?	From 60 minutes to 90 minutes.
**22**	Data saturation	Was data saturation discussed?	Yes
**23**	Transcripts returned	Were transcripts returned to participants for comment and/or correction?	No
**Domain 3: analysis and findings**			
Data analysis			
**24**	Number of data coders	How many data coders coded the data?	Two
**25**	Description of the coding tree	Did authors provide a description of the coding tree?	Yes
**26**	Derivation of themes	Were themes identified in advance or derived from the data?	Derived from the data
**27**	Software	What software, if applicable, was used to manage the data?	Microsoft excel and NVivo 12 (QSR International, Doncaster, Australia)
**28**	Participant checking	Did participants provide feedback on the findings?	No
Reporting			
**29**	Quotations presented	Were participant quotations presented to illustrate the themes /findings? Was each quotation identified? e.*g. participant number*	Yes
**30**	Data and findings consistent	Was there consistency between the data presented and the findings?	Yes
**31**	Clarity of major themes	Were major themes clearly presented in the findings?	Yes
**32**	Clarity of minor themes	Is there a description of diverse cases or discussion of minor themes?	Minor themes did not emerge from the data.
