# Supplementary material for: A Patient-Centred Medical Home Care Model for Community-Dwelling Older Adults in Singapore: A Mixed-Method Study on Patient’s Care Experience
Source: Int J Environ Res Public Health. 2022 Apr 14;19(8):4778. doi: 10.3390/ijerph19084778 (PMC9030670; doi:10.3390/ijerph19084778)
Supplement: Supplementary file 1 [file ijerph-19-04778-s001.zip › Supplementary File S2.FGD Topic Guide.pdf]

Supplementary File S2: Focus Group Discussion Topic Guide

1. Opening

1.1. Could you tell us a little about yourself?

Prompt:

- How is your usual day like?
- On a daily basis, who takes care of you?

1.2. Where do you usually go to seek advice first about your health and well-being?

Prompt:

- Who would you define as your primary care provider (*to prompt for the main healthcare provider for their chronic care for the past 6 months*)?
- What are the usual reasons why you go to your primary care provider?

2. Experience at PCMH clinic and/or care management

2.1. How did you become a patient of PCMH clinic?

2.2. Thinking about the first time that you found out about PCMH clinic and/or their care management program till now, could you share your experience as a patient of the clinic and/or the care management program?

2.3. How would you compare the care you receive as a patient of PCMH Clinic and/or care management to your previous primary care provider (before PCMH)?

3. Patient-provider partnership and communication

3.1. How was your experience with the staff (e.g. doctor, nurses, care manager, admin staff) in the clinic?

3.2. What information were you given when you were treated in the clinic regarding …

Prompt:

- Your condition?
- Your medication?
- Self-management (e.g. patient education) or follow-up care at home?
- Social support from the clinic/care manager?

4. Comprehensiveness

4.1. Do you feel that the clinic provides services that fulfil your needs (e.g. physical, mental and social)? Why do you say so?

5. Whole person orientation (patient-centred)

5.1. Please describe your relationship with the clinic staff and/or care manager?

Prompt:

- What are the things that the clinic staff and/or care manager and you talk about?
- What other things (apart from health-related issues) that the clinic staff and/or care manager and you the clinic staff talk about?

5.2. To what extent do you think the clinic staff know and understand about your current medical conditions (e.g. your problems, medical history, preference in treatment options and care)?

5.3. How would you describe the communication that your family member(s) have with the staff of the clinic and/or care manager?

Prompt:

- How often does the clinic staff talk and discuss about opinions of family members on your treatment and care?

- What were the information given to your family member on your treatment options and care?

5.4. How confident are you in managing your own condition at home? Why or why not?

Prompt:

- What are the facilitators, challenges and barriers of you having confidence in managing your condition at home?
- How would you describe the support that the clinic gives you in managing your own care at home?

6. Coordination/integration of care

6.1. Please share with us the reason(s) that was given by the clinic on why you need to go to different places for your treatment and care? (e.g. hospital, specialist, social care and community services, etc.)

6.2. How do you usually arrange for an appointment to the different providers?

Prompt:

- Does someone from the clinic help you in arranging the appointment?
- How long is usually the waiting time?

6.3.How would the clinic follow-up on your condition after that?

7. Enhanced access

7.1 How do you usually get the care you need during weekend, public holidays, and after office hours?

Prompt:

- What information were given to you by the clinic about who to call or go to for your treatment and care during weekend, public holiday, after office hours and during an emergency?

7.2. How is your experience in appointment booking in this clinic?

- How likely is it to have the same day appointment for your care?
- How long is usually the waiting time in the clinic?

8. Impacts/Outcomes

This section pertains to changes that you may have experienced after you received care from the clinic/care management.

8.1 How would you describe the changes in your **health and lifestyle since you started to visit the clinic / care management**? (i.e. disease-related, psychological-related and social health-related condition)

Prompt:

- What do you think contributed to these changes?
- Were there any changes in the frequency that you use healthcare services (e.g. specialist clinic and acute hospital)?

8.2 What suggestions would you have to improve the clinic/care management care delivery?

Prompt:

- What are some aspects of the care delivery in the clinic that was done well?
- What aspects of the clinic/care management would you like to see improve?
- What would enable you to have a better experience of care?

Closing

Before we end today's discussion, does anyone has any final comments/thoughts to share?
