# Supplementary material for: A Patient-Centred Medical Home Care Model for Community-Dwelling Older Adults in Singapore: A Mixed-Method Study on Patient’s Care Experience
Source: Int J Environ Res Public Health. 2022 Apr 14;19(8):4778. doi: 10.3390/ijerph19084778 (PMC9030670; doi:10.3390/ijerph19084778)
Supplement: Supplementary file 1 [file ijerph-19-04778-s001.zip › Supplementary File S3. Sensitivity analysis table.pdf]

Supplementary File S3. Sensitivity analysis table

Table S1. Comparison of Consumer Assessment of Health Providers and Systems Clinician & Group Survey (CG-CAHPS), Adjusted Mean Composite Scores between usual care and PCMH – Sensitivity analysis on participants with baseline survey done within 60 days vs those outside 60 days from PCMH enrolment date.

Measures	Complete cohort		Sensitivity analysis	
	**Usual Care (N=184), adjusted mean score[1]**	**PCMH (N=166), adjusted mean score[2]**	**Usual Care (N=183), adjusted mean score[3]**	**PCMH (N=165), adjusted mean score[4]**
**Composite measures**				
Timely care access[5] – 3 questions asking about patient's experience to get timely appointments for urgent and routine care needs, and timely answers to medical questions	-	-	-	
Patient-provider communication[6] – 4 questions asking how often providers explained things clearly, listened carefully to, showed respect for and spent enough time with patient	3.6085	3.9284	3.6103	3.9297
Care coordination[7] – 3 questions asking how often provider seemed to know patient's medical history, followed-up to give results of blood test, and asked about all the prescription medications being taken	3.6155	3.8074	3.6207	3.8097
Helpful, courteous, and respectful office staff[8] – 2 questions asking how often office staff were helpful and treated patient with courtesy and respect	3.5597	3.8968	3.5628	3.8963
Support for patients in caring for their own health[9] – 2 questions asking whether someone from provider's office discussed with patient his or her specific goals for health and whether there were things in life that make it hard for patient to take care of his or her health	0.1535	0.3167	0.1541	0.3190
**Rating measure**				
Patient's overall rating of the provider[10] – 1 question asking patient to rate his or her provider on scale of 0 (the worst provider possible) to 10 (the best provider possible)	7.2616	8.3974	7.2491	8.3853

[1,2,3,4] The CAHPS Analysis Program adjusted the survey data for participant's age and general health rating before the score was calculated, to account for case-mix difference. [5] Fewer than 100 responses, score not analysed. [6,7,8] Each question has response categories in a 4-point scale – never, sometimes, usually and always, scored as 1, 2, 3 and 4, respectively.

[9] Each question has response category in a 2-point binary scale – no and yes, scored as 0 and 1, respectively.

[10] Question is based on response with a 11-point scale – 0 to 10.
